# Cadmium Toxicity and Health Effects—A Brief Summary

**DOI:** 10.3390/molecules28186620

**Published:** 2023-09-14

**Authors:** Angelika Edyta Charkiewicz, Wioleta Justyna Omeljaniuk, Karolina Nowak, Marzena Garley, Jacek Nikliński

**Affiliations:** 1 Department of Clinical Molecular Biology, Faculty of Medicine with the Division of Dentistry and Division of Medical Education in English, Medical University of Białystok, 15-269 Białystok, Poland; 2 Department of Analysis and Bioanalysis of Medicines, Faculty of Pharmacy with the Division of Laboratory Medicine, Medical University of Białystok, 15-222 Białystok, Poland; 3 Department of Obstetrics and Gynecology, C.S. Mott Center for Human Growth and Development, School of Medicine, Wayne State University, Detroit, MI 48201, USA; 4 Department of Immunology, Faculty of Pharmacy with the Division of Laboratory Medicine, Medical University of Bialystok, 15-269 Bialystok, Poland

**Keywords:** cadmium, exposure and absorption, toxic element, poisoning and effects

## Abstract

Cadmium (Cd) is a ductile metal in the form of a blueish or silvery-white powder. It is naturally found in soil (about 0.2 mg/kg), minerals, and water. Cd belongs to the group of toxic, carcinogenic, and stimulating elements. Its biological half-life in the human body ranges from 16 to even 30 years on average. Some lung diseases (such as emphysema, asthma, and bronchitis) and high blood pressure are thought to be related to slow poisoning. The symptoms of cadmium poisoning may vary depending on the time of exposure, the type of diet, and the age and health status of the exposed people. For non-smokers and non-occupational exposures, the only source of exposure is diet. The FAO/WHO recommends that the tolerable cadmium intake for an adult is approximately 0.4–0.5 mg/week (60–70 µg per day). Cadmium is primarily absorbed through the respiratory system (about 13–19% of Cd from the air), but it can also enter through the digestive system (about 10–44%), when dust is mixed and swallowed with saliva. The amount of accumulated Cd ranges from 0.14 to 3.2 ppm in muscles, 1.8 ppm in bones, and 0.0052 ppm in the blood. People who are most frequently exposed to heavy metals should be continuously monitored in order to maintain a healthy lifestyle, as well as to implement effective preventive measures and improve public health.

## 1. Introduction

Cadmium (Cd) is a malleable metal in the form of a blueish or silvery-white powder. It easily reacts with other substances that are most commonly used in cells and batteries including nickel–cadmium batteries, alloys, pigments, plastic stabilizers, dyes, and paints, as well as in glass manufacturing and galvanic industry [1,2,3,4,5,6,7,8,9]. This element was discovered by F. Stromeyer in Göttingen, Germany, in 1817 [1]. Cd is used in nuclear reactors as a regulator of the uranium fission reaction by electron capture [3]. It occurs naturally in soil, minerals (sulfides, sulfates, carbonates, chlorides, and hydroxide salts), and water [2,4]. Cd is obtained as a by-product of zinc production from ZnS (zinc sulfide), although it is most often found together with zinc, lead, or copper due to their similar properties [3,10]. The greatest exposure to Cd occurs in the metallurgical industry (in zinc smelters or in units where pig iron is purified) [2,11,12].

Around 600 million people are annually affected by a contaminated environment. Contamination of food with heavy metals is more common in polluted agricultural regions, posing a very serious problem worldwide [13,14]. It is also estimated that more than 13% (about 0.24 billion hectares) of the world’s total arable land and about 40% of lakes and rivers are contaminated with heavy metals [14]. The continued use of Cd in industry drastically affects the environment, resulting in high exposure of humans to the element [15].

In recent years, the biological role of Cd has been widely investigated, as it belongs to the group of toxic, carcinogenic, and stimulating elements. It is known that the content of Cd in the human body varies depending on the location [1,7,9].

The biological half-life of Cd in the human body ranges from 16 to even 30 years [16,17]. It is believed that some chronic lung diseases (such as emphysema, asthma, and bronchitis) and high blood pressure are related to slow poisoning by Cd in small doses [10,11,18,19]. Moreover, long-term exposure to Cd can lead to various diseases, such as cancer, leukemia, and genetic toxicity [7,8,9]. Numerous studies have confirmed that exposure to heavy metals, even at low levels, can cause serious damage to human organs. Acute ingestion can result in abdominal pain, burning sensations, nausea, vomiting, salivation, muscle cramps, dizziness, shock, unconsciousness, or even convulsions within 15–30 min [20,21]. Recent epidemiological data indicate that Cd exposure may also be associated with some cancers (prostate, bladder, pancreatic, kidney, and breast). It may play a role in the development of diseases related to the central nervous system (CNS), such as Alzheimer’s disease (AD), Parkinsonism and Parkinson’s disease (PD), Huntington’s disease (HD), amyotrophic lateral sclerosis (ALS), and multiple sclerosis (MS), or in the deterioration of cognitive and behavioral functions, as well as chronic diseases, such as osteoporosis and osteomalacia of pelvic bones, femurs, vertebral bodies, and bones of the shoulder blades. It can cross the placenta and the barrier to the fetus, exerting teratogenic effects, and is associated with Itai-Itai disease, cardiovascular disease, lung function abnormalities, damage caused in the kidneys, etc. [2,3,9,18,22,23,24,25,26]. The kidneys are the main target organ and the most sensitive to Cd contamination and a reduced glomerular reabsorption rate [2].

The purpose of this review is to describe the toxic effects of Cd in the human body. The paper also summarizes the sources and levels of Cd contamination, emphasizing that its environmental exposure varies depending on the length of exposure.

## 2. Sources of Cadmium

The main source of cadmium is stack dust, which is generated during zinc purification by distillation and, due to its high volatility, is deposited in all fractions [10]. Cd is mainly used for coating other metals, mainly steel, or as an anticorrosion coating of steel sheets [3,8,10]. It acts as a very good protective coating in an alkaline environment and is also commonly used to produce low-melting alloys, such as Wood’s metal, used in fire protection systems [9,10]. Currently, in Poland, heavy industries are practically disappearing, and therefore the main routes of Cd exposure in the country are cigarette smoking and the consumption of contaminated food [15,27]. Additionally, Tchounwou et al. reported the same routes of Cd exposure in the US [9]. In addition to the consumption of contaminated food and smoking, people can be exposed to cadmium in different ways, including employment in the metal industry or working in cadmium-contaminated sites [27,28,29].

## 3. Exposure and Accumulation of Cadmium in the Human Body—Pathological Effects

The symptoms characteristic of Cd poisoning vary depending on the length of exposure, the type of diet, and the age and health status of those exposed. The effect of Cd on the body can be modified by its interactions with other metals, such as zinc, selenium, copper, iron, and manganese. Tobacco smoke acts synergistically, very quickly interacting with this element [3,9,17].

**Figure 1:**
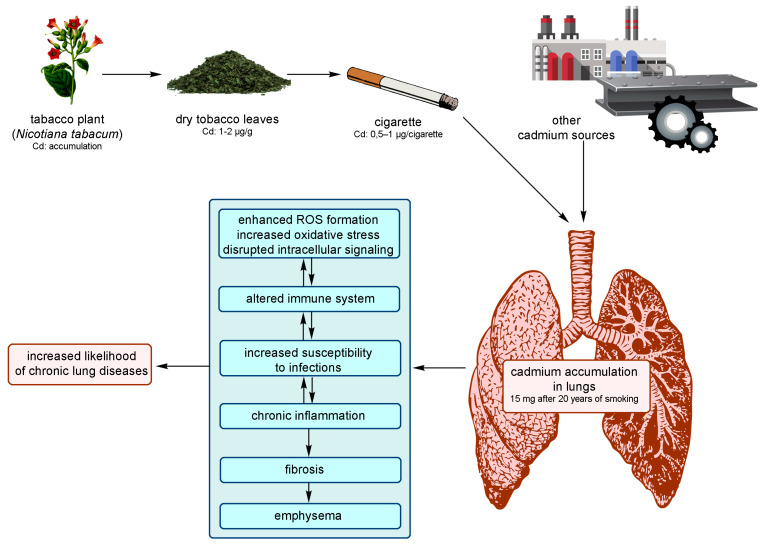
Cadmium exposure leading to the development of smoking-related lung diseases.

**Figure 2:**
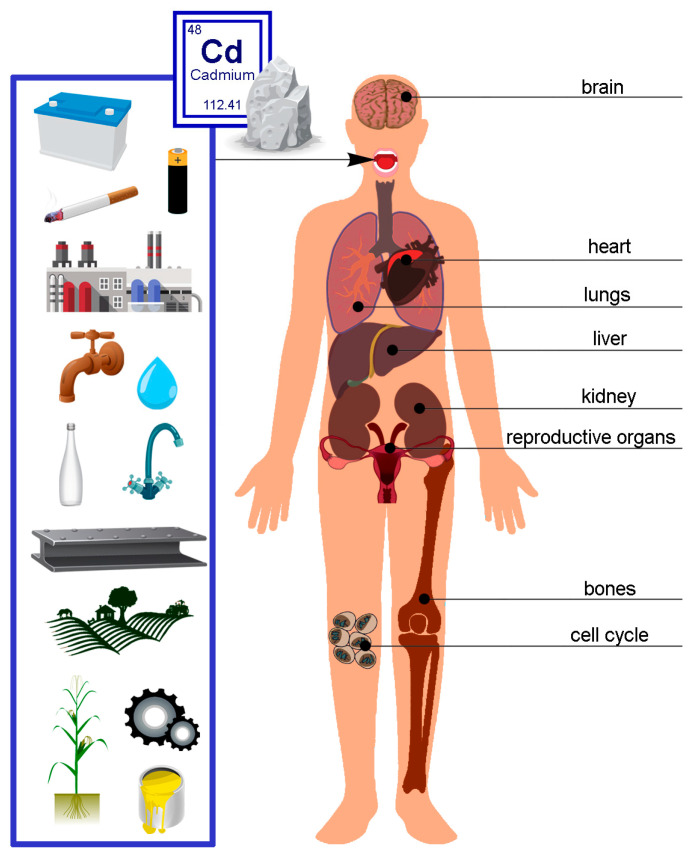
Sources of cadmium and its most significant effects on different parts of the human body.

Cadmium is mainly absorbed during inhalation, partly through the digestive system when particles of dust are swallowed with saliva [6,7,9]. This element accumulates (Table 1) most often in the lungs, liver, kidneys, pancreas, testicles, muscles, adipose tissue, and skin, inhibiting the activity of sulfur-containing enzymes (Figure 1 and Figure 2) [2,3,9,10,17,22,30]. Figure 1 illustrates some of the most common sources of Cd and how Cd exposure leads to the development of lung diseases. It also demonstrates the health effects associated with the long-term accumulation of Cd in the lungs. It is estimated that about 13–19% of Cd is absorbed into the lungs from the air, and about 10–44% through the digestive tract, mainly into the small intestine. Cadmium binds to red blood cells in complexes with large-molecule proteins (albumin) and accumulates in the liver, while when complexed with small-molecule proteins (metallothionein—MT) it is reabsorbed in the renal tubules [3,5,22,30]. The amount of Cd accumulated ranges between 0.14 to 3.2 ppm in muscles, 1.8 ppm in bones, and 0.0052 ppm in the blood [1]. Figure 2 illustrates some of the most common sources of Cd and how exposure to Cd leads to the development of many diseases or pathologies in the body. It also shows the health effects associated with the long-term accumulation of Cd in organs/tissues and its reaction in the body.

### 3.1. Pathological Effects in the Respiratory System

In the respiratory system, Cd can cause irritation to the mucous membranes of the nose (impairing the sense of smell) and the upper respiratory tract. Occupational-exposure-related poisoning, mainly in the metallurgical industry, occurs as a result of the absorption of fumes generated during the welding, melting, or soldering of cadmium-containing materials (Figure 2) [3,11]. Changes in the respiratory system can be detected by laryngological examination, spirometry, and chest X-ray [3]. The initial symptoms of poisoning are similar to metallic fever and pulmonary edema, which may occur within 24 h. Acute Cd poisoning occurs due to exposure at a concentration of 0.5 mg/m^3^ from fumes and 3 mg/m^3^ from the respirable fraction of dust. It usually results in chronic bronchitis, but people working in the metallurgical industry report an impaired sense (or even complete loss) of smell, the drying of nasal mucous membranes, or their ulceration. It is also common to have a dry cough at first, followed by expectorant symptoms of chronic bronchitis. Probable emphysema related to cadmium exposure is manifested by exertional dyspnea, decreased exercise tolerance, and reduced lung ventilation efficiency [3,22,31]. Chronic inhalation of Cd particles is associated with lung function abnormalities and chest radiographs that are consistent with emphysema, while occupational exposure to airborne particles is associated with decreased olfactory function [9].

### 3.2. Pathological Effects in the Nephrological System

The extent of damage caused in the kidneys by Cd (Figure 2) depends on the level of accumulation of cadmium MT in the organ, with changes mainly observed in the primary tubules along with early signs of proteinuria [3]. It is worth emphasizing that acute oral poisoning is no longer encountered in occupational exposure conditions. Meanwhile, chronic poisoning develops after a few or several years of exposure to Cd, or even after the exposure has ceased. Long-term exposure to Cd in the past is associated with the presence of Cd in the urine (Cd-U) at a detectable concentration, which (usually calculated in relation to creatinine) indicates accumulation, i.e., Cd burden on the kidneys. Symptoms of Cd burden can involve one or more organs, with the kidneys being the most affected. In the exposed individuals, the critical concentration of Cd in the renal cortex may even reach 200 mg/kg, while in the urine, it ranges from 0.889 µmol/l (10 µg/g creatinine) to 1.333 µmol/l (15 µg/g creatinine). The acceptable biological concentration of Cd in the urine is 0.0445 µmol/l (5 µg/g creatinine) [3,7]. It is estimated that about 2.3% of the US population have elevated levels of urine cadmium (>2 µg/g creatinine) [9]. Proteinuria may be one of the longer-lasting symptoms, followed by glycosuria, aminoaciduria, increased excretion of calcium and phosphorus in the urine, and increased creatinine concentration. Toxic nephropathy is often the only consequence of cadmium exposure [3,7,22,31].

Cd is excreted from the human body very slowly, mainly in the urine, feces, saliva, or sweat, posing serious risks [10]. Three stages of urinary cadmium excretion can be distinguished [3]:

– Stage 1: Cadmium accumulates in the renal cortex by binding to MT. The concentration of Cd excreted in the urine is proportional to the content of this element in the kidneys; its concentration in the urine reflects past exposure.
– Stage 2: With high exposure and depletion of MT stores in the kidneys, the urinary cadmium concentration reflects both current and past exposure.
– Stage 3: When the renal tubules are damaged, the kidneys lose the ability to reabsorb cadmium, which results in significant urinary excretion of this element; this reflects ongoing exposure and its elimination from the kidneys.

### 3.3. Pathological Effects in the Circulatory System

Cadmium plays a key role in select cardiovascular diseases associated with smoking, such as peripheral arterial disease and ischemic heart disease (Figure 2). Chronic exposure to Cd can lead to arterial hypertension, atherosclerosis, and impaired heart function [9,45]. The presence of cadmium in the blood reflects its recent exposure (e.g., through smoking or in the workplace) [9]. However, the effect of Cd on the cardiovascular system remains controversial, as some studies have shown that at extremely low doses this element can adversely affect the system [46]. Li et al. (2019) showed an association between Cd exposure and the cardiovascular disease pathway. The authors also explained that the development of some of the cardiovascular diseases caused by smoking is mediated by cadmium [31]. The currently acceptable biological limit of blood cadmium is 5 μg/L according to the American Conference of Governmental Industrial Hygienists (ACGIH, 2010; https://www.acgih.org/, accessed on 10 September 2010, ACGIH) and 2.7 μg/L according to Neumeister et al. [47]. Meanwhile, our previous research showed that occupational exposure to Cd can reduce the blood level of selenium in men who are employed in the metallurgical industry and who also suffer from cardiovascular diseases. A statistically significant positive correlation between lead (Pb) and Cd has been observed, indicating a mixed exposure [11]. Studies from Sweden confirmed the relationship between exposure to cadmium and the development of atherosclerosis [32] and ischemic stroke [48], while subsequent studies showed only further changes in the circulatory system with prolonged exposure to heavy metals [49,50].

### 3.4. Pathological Effects in the Skeletal System

Because cadmium interferes with the metabolism of calcium, magnesium, iron, zinc, and copper in cells, it causes bone demineralization, osteomalacia, and osteoporosis, as well as disturbances in the regulatory functions in which these ions are involved (Figure 2) [9,33]. One of the effects of chronic diseases, such as osteoporosis and osteomalacia of pelvic bones, femurs, vertebral bodies, and bones of the shoulder blades, is persistent pain in the spine, pelvis, and limbs, which can be detected in a radiological examination [3,22,31]. A population-based study (Women’s Health in the Lund Area, WHILA) of women aged 50–59 years in southern Sweden (*n* = 10.766) confirmed a clear link between increased Cd burden and decreased bone mineral density (BMD), increased bone resorption of urinary deoxypyridinoline (U-DPD), and decreased levels of parathyroid hormone (PTH) [51]. Staessen et al. [52] showed that the effect of Cd on bone resorption was even more pronounced after menopause (interaction), which is in line with the evidence indicating postmenopausal women as the main population affected by Itai-Itai disease [53].

One of the most widely recorded diseases caused by exposure to Cd is Itai-Itai disease. This disease was more common in Toyama, Japan, where people ate rice grown in irrigation water contaminated with the element [2,4,7,17,51]. It is manifested by pain in the bones and joints, a specific waddling gait due to bone distortion, and susceptibility to complex fractures in the joints [3,10]. The changes in the skeletal system caused by this disease may be related to the inhibition of the function of primary renal tubular cells, which reduces the efficiency of vitamin D metabolism, and thus calcium absorption, ultimately leading to bone demineralization [3].

### 3.5. Pathological Effects in the Reproductive System

With very slow transport, Cd can cross the placenta and the barrier to the fetus, exerting teratogenic effects (Figure 2) [5,7,16,17,18]. Most of the element is retained in the tissue. However, there is insufficient data on its role in early pregnancy loss [18] and its direct impact on fetal development in humans [33]. The study by Omeljaniuk et al. [18] confirmed that the content of Cd and lead in women with spontaneous abortion was significantly higher in the blood (2.730 ± 2.07 µg/L) and much higher in the placenta (214.4 ± 514 ng/g) compared to the control group (0.166 ± 2.523 µg/L and 127.4 ± 85 ng/g, respectively) [18]. It is worth noting that women have higher concentrations of Cd in the blood, urine, and kidneys than men, and this can be attributed to the depletion of the body’s iron supply and overt iron deficiency, which are common in women of reproductive age [54]. Furthermore, the milk of smoking mothers may contain twice as much Cd as that of nonsmokers [33]. Meanwhile, a study of 163 pregnant women from Recôncavo Baiano, Brazil, in which materials collected from blood, toenails, and hair were analyzed, indicated that as many as 61.1% had elevated blood Cd levels. A conducted binary logistic regression showed that factors such as low socioeconomic status, burning household waste, inactive smoking, having many children, and home renovations significantly increased the likelihood of having high levels of other heavy metals as well [55]. The study by Kasperczyk et al. revealed that the number of normal sperm forms decreases with increasing Cd concentration in the blood [34]. The toxic effects of cadmium primarily impair testicular function through damage to the vascular endothelium, Leydig and Sertoli cells, and intercellular connections, and necrosis of the seminiferous tubules, which inhibits testosterone synthesis and impairs spermatogenesis. Cadmium also disrupts the function of the prostate gland, leading to changes in its hormonal and secretory functions, which impairs male fertility [33].

### 3.6. Pathological Effects in the Nervous System

The neurotoxic effect of cadmium is still unclear. Cadmium can adversely affect the human nervous system when its concentration exceeds >0.8 μg/L in the urine and 0.6 μg/L in the blood. Such high concentrations are currently recorded in industrialized countries, so it can be concluded that environmental exposure to this metal can pose a threat to the nervous system. Cadmium may also be involved in the etiopathogenesis of neurodegenerative diseases (Figure 2). It is believed that Cd may play a role in the development of diseases related to the central nervous system (CNS), such as Alzheimer’s disease (AD), Parkinsonism and Parkinson’s disease (PD), Huntington’s disease (HD), amyotrophic lateral sclerosis (ALS), and multiple sclerosis (MS), or in the deterioration of cognitive and behavioral functions [6,25,26]. Earlier studies have shown a higher concentration of Cd in the hair of children suffering from neurological disorders, learning disorders and/or behavioral difficulties, neuropathy, and impairments in memory, attention, and psychomotor functions [25,35,36]. Since Cd can interfere with nervous system function in children, even at much lower concentrations (>0.38 μg/L in blood and >0.1802 μg/L in urine), it may be considered as one of the potential contributors to these disorders in this population [6,25].

### 3.7. Cell Cycle

The influence of cadmium on energy transformations in the cell cannot be unequivocally assessed. However, it has been found that Cd inhibits tricarboxylic acid cycle enzymes and stimulates glycolysis. The element also inhibits many enzymes involved in DNA synthesis and the active transport of sodium and potassium, induces lipid peroxidation, disrupts carbohydrate (glutathione) metabolism, and inhibits tissue respiration (Figure 2) [2,3,9,18]. Cadmium has the ability to interact with zinc, copper, iron, magnesium, calcium, and selenium ions present in the cells, where these ions fulfill important biological functions. Thus, it induces metabolic disorders in cells, ultimately resulting in morphological and functional changes in several organs. Short-term exposure to Cd increases the activity of superoxide dismutase (SOD), catalase (CAT), and peroxidase and glutathione reductase (GSHPx and GSHR), which leads to the activation of defense mechanisms, subsequently inducing the adaptive response of cells. With prolonged exposure, cadmium causes a marked decrease in cell activity. The mechanism behind this toxic action is the induction of oxidative stress in cells, which results in peroxidative damage to cell membranes [9,33].

### 3.8. Carcinogenic Effect of Cadmium

Due to its cytotoxic effects, Cd may act as a carcinogen when inhaled; however, there is insufficient evidence showing carcinogenic activity resulting from its oral ingestion. Cd can induce apoptotic or necrotic events (group I of the International Agency for Research on Cancer (IARC) classification) [7,9,17,28,56,57]. Environmental or occupational exposure to this element is most often associated with cancers of the lungs, breasts, prostate, pancreas, urinary bladder, and nasopharynx (Figure 2) [58]. Systemic or direct exposure to Cd can also cause proliferative changes in the prostate gland, including adenocarcinomas [9]. Some studies suggest a potential link between Cd exposure and breast cancer mediated by epigenetic changes [44]. Cadmium is a weak mutagen compared to other carcinogenic metals [9]. Its main carcinogenic mechanism includes the activation of inflammatory processes, generation of reactive oxygen species (ROS), epigenetic changes, attenuation of apoptosis, DNA damage, impairment of DNA repair, oxidative stress, changes in gene expression, and abnormal DNA methylation [4,9,17,33]. Oxidative stress plays a key role in the toxicity of this element [9,37]. A proper DNA repair system corrects errors caused by metabolism and environmental carcinogens; however, the impairment of DNA repair causes the accumulation of damaged DNA, which promotes cancer [4,9,59]. Cadmium can inhibit DNA repair processes, including nucleotide and base excision and their further mismatch [9,38]. The loss of the DNA repair mechanism allows cells with damaged DNA to accumulate, which can cause carcinogenic mutations [17]. Some studies have shown a relationship between total cancer and lung cancer in relation to lifelong environmental exposure to cadmium [9,23,39]. However, a study in the Chaoshan population of southeast China showed an association between the presence of Cd in the blood and nasopharyngeal carcinoma (NPC), in which the median blood Cd concentration in the cases was significantly higher than that in the control group. It was also confirmed that smokers had a higher level of Cd load [40]. Thus, Cd appears to be a risk factor for NPC, and chronic exposure to the element may promote the onset and development of this type of cancer. Recent epidemiological data indicate that Cd exposure may also be associated with prostate cancer [7,24], bladder cancer [41], pancreatic cancer [42], and kidney cancer [7,43].

## 4. Environmentally Degrading Effects of Cadmium

Cadmium is a very rare element, but it is classified as a harmful environmental pollutant [10]. It should be emphasized that Cd does not decompose in the environment, nor is it easily removed from the soil, where it is often introduced through phosphate fertilizers (e.g., superphosphates) [5,7,8,33]. Its high level in the water, air, and soil is usually a consequence of industrial activity [4,7]. It has been shown that in Poland, rail transportation is one of the sources of toxic substances in the soil, both in the vicinity of junctions and along railway routes, which is often related to the presence of anticorrosion paints, brake pads, lubricating oils, and fuels, as well as the impregnation of railway sleepers [2]. A Chinese study indicated that low Cd concentrations in rice can be attributed to the poor effects of some major pollution activities, including mining, irrigation, and the use of chemical fertilizers and pesticides [60,61].

A study by Wieczorek et al. [62], which analyzed the ecological risk of Cd and Pb in the soil in the region of Małopolska in Poland, showed a large variation in the accumulation of these elements, depending on both natural and anthropogenic factors. Significantly higher pollution was found in areas where mining and metallurgical activities were carried out [63]. Some authors recommend the remediation of soils contaminated with heavy metals, as it can reduce the associated risks, increase the availability of land resources for agricultural production, and thus improve food security, as a result of changes in the use of land. Soil washing and phytoremediation are the best available technologies for treating heavy-metal-contaminated soils, but are often practiced only in developed countries. However, these technologies are also recommended in developing countries, where agriculture, urbanization, and industrialization contribute to environmental degradation [14,20].

**Figure 3:**
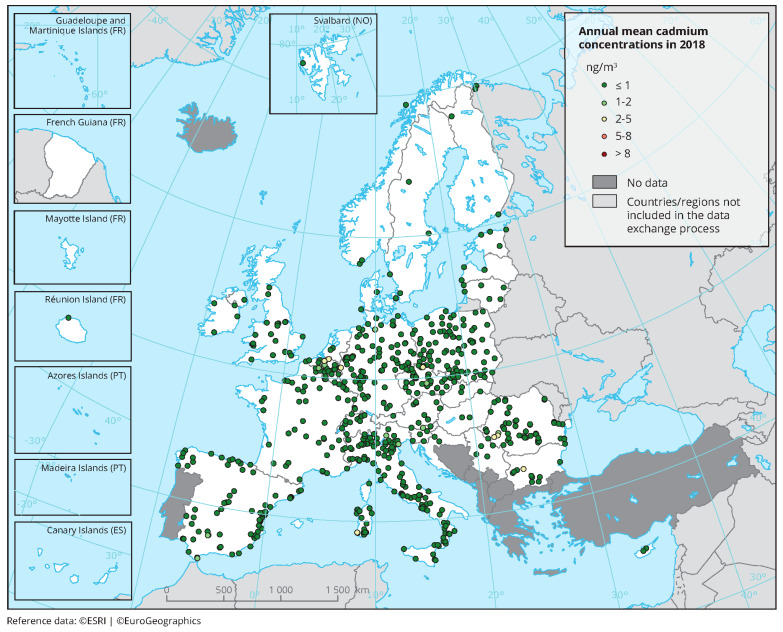
Annual mean Cd concentrations in 2018 in Europe [64].

In 2018, the European Environment Agency reported Cd concentrations (Figure 3). Only stations reporting more than 14% of valid data were included. In 2018, the European Environment Agency presented data on contamination with concentrations of Cd in select European countries (Figure 3) [64].

**Figure 4:**
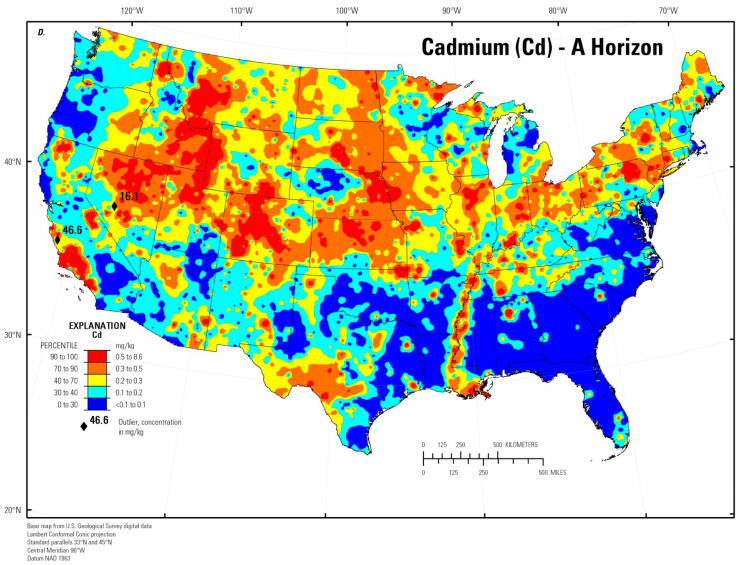
Cadmium in topsoil (called A Horizon) across the United States [66,72]. Distribution of cadmium (Cd) in the soil A Horizon, conterminous United States (LLD—lower limit of determination; number below LLD—1054; MAD—median absolute deviation (ND, not determined); CV—coefficient of variation (ND, not determined); mg/kg—milligrams per kilogram) [66].

The map from the United States (Figure 4) shows the distribution of cadmium in topsoil (called A Horizon) across the country (from blue, less than 0.1 ppm, to red, more than 0.5 ppm). Soil samples (*n* = 4.841) were taken from a depth of 0 to 5 centimeters and Cd concentrations were measured in milligrams per kilogram. It is worth noting that the southeast has lower levels than the Great Plains and Rocky Mountain regions. Scientists have very clearly warned that determining the amount of Cd in the soil is only the starting point for developing mitigation strategies. Very important are the identification of soil characteristics, crop genetics, and agricultural management techniques themselves. It should also be noted that some crops high in iron and zinc also tend to absorb Cd, and that there is a need to better understand the mechanisms involved in Cd bioaccumulation in different plant species and different soil environments [65,66].

In the case of existing heavy metal (including Cd) contamination of soils, it is necessary to use them appropriately for agricultural purposes, while for land with very heavy contamination, a change in the form of its use, such as remediation or reclamation (cleaning) is carried out in order to restore the degraded land to its usable value. Remediation may include self-purification through the removal of contamination (up to an acceptable content of risk-causing substances in the soil) and actions leading to the removal of significant risks to human health and the environment, taking into account the current or planned use of the site (reducing the amount of contamination; limiting its potential to spread and controlling it through periodic soil testing over a specified period of time; carrying out self-cleaning of the land surface with support for self-purification; limiting human access to the contaminated site; the possible need to change the use of the contaminated site). The second method, i.e., reclamation, includes biological, chemical, and electrochemical techniques leading to the partial removal of Cd from the soil, which are also very expensive (except for biological techniques) and do not guarantee the complete purification of the soil. Unfortunately, it is very damaging to the soil [67,68].

Soil reclamation methods:

– transformation of heavy metals into forms inaccessible to plants (soil liming; increasing the amount of organic matter; vaping with a combination of organic matter);
– partial removal of heavy metals from the soil (phytoremediation; introduction of natural and synthetic adsorbents into the soil; cultivation of industrial crops; extraction and flushing of the soil; removal of contaminated soil);
– a complete change in the form of land use (cultivation of shrubs and trees) [67,68].

In the population of workers exposed by inhalation, there was a statistically significant increase in lung cancer mortality in early studies, although this was not confirmed in later studies [69]. It should be noted that the JECFA (Joint FAO/WHO Expert Committee on Food Additives and Contaminants) has identified the kidney as critical for cadmium toxicity [2,5,70,71]. The Scientific Committee on Occupational Exposure Limits (SCOEL) (2010) recommends an occupational exposure level (OEL) of 4 µg Cd/m^3^ (inhalable fraction) as a measure of protection against long-term local effects (respiratory effects, mainly lung cancer). Information is based on human data showing changes in residual lung volume at a cumulative CdO vapor exposure of 500 µg Cd/m^3^ × years, which corresponds to a 40-year exposure of 12.5 µg Cd/m^3^ (LOAEL—lowest-observed-adverse-effect level). Thus, applying an uncertainty factor of 3 (LOAEL to NOAEL—no-observed-adverse-effect level) leads to a value of 4 µg/m^3^ [69]. A European risk assessment report proposed a LOAEL of 2 μg Cd/g creatinine; however, in an evaluation of the risk assessment document, it was concluded that the effects may occur even at lower levels (as low as 0.5 μg/g creatinine) [2,5,7,8]. Satarug et al. [70] made it very clear that the current nephrotoxicity thresholds for cadmium are outdated and do not protect human health. In this case, the health risk assessment should also be based on current data that are adequate for the region, exposure, age, group, gender, etc.

## 5. Accumulation of Cadmium in Tobacco Smokers

Cadmium is known to be significantly absorbed in cigarette smoke because it naturally accumulates in the tobacco plant (*Nicotiana tabacum*) [2,12,45]. Smoking 20 cigarettes per day causes the increased absorption of cadmium [3], and the content of cadmium in tobacco leaves ranges from 1 to 2 μg × g^−1^ of dry matter, which is equivalent to 0.5–1 μg of cadmium per cigarette [9,12,73]. The average daily Cd intake from 20 cigarettes is about 1 μg. However, it is assumed that cadmium intake from smoking can be reduced by changes in tobacco processing and cigarette design [27]. Long-term cigarette smoking (e.g., 20 years) introduces approximately 15 mg of cadmium into the smoker’s body [33]. Studies have shown that the blood Cd concentration in tobacco smokers is several times higher than that of non-smokers [4,7,15,74]. It may also be related to the nature of tobacco plants, which accumulate relatively high concentrations of Cd in tissues, especially in the leaves [4,27]. According to the authors, the current body of evidence does not clearly distinguish the effects of cadmium in the lungs from the effects of other potentially pathogenic compounds in tobacco smoke. Local cadmium accumulation in the lungs is most common among long-term smokers. This is critical, considering that the biological half-life of cadmium in the human body is up to 30 years, implying the possibility of significant Cd retention in the lungs of this population. Cadmium accumulated in the lungs and other organs may inevitably affect intracellular signaling, resulting in an impaired host defense function (including innate immunity). This may contribute to increased susceptibility to bacterial infections leading to chronic inflammation, fibrosis, and emphysema among long-term smokers [73]. Several recent studies confirm the development of lung diseases related to both short- and long-term exposure to smoking [9,12,27,58].

## 6. Accumulation of Cadmium in Food

Since the diet is also a major source of Cd, the European Food Safety Authority (EFSA) in cooperation with FAO/WHO experts has published standards and guidelines for the safe intake of this element for both adults and groups with increased susceptibility, such as children. In order to correctly assess long-term or short-term exposure, the health risks associated with Cd exposure should be analyzed, the total or average intake should be assessed over several months, and the tolerable intake should be assessed for approximately one month or more. [2,5,69,70]. The EFSA estimates that children (mainly infants and young children) have a higher relative intake of Cd than adults [65]. It should be mentioned that providing adequate food with micronutrients (Zn, Fe, Ca) can protect against the absorption and toxicity of cadmium [5]. The daily Cd intake may range from 0.007 to 3 mg, while the toxic dose of this element ranges from 3 to 330 mg and the lethal dose from 1.5 to 9 g (for fumes, from 2.600 mg/m^3^ to 2.900 mg/m^3^ × minute of exposure). The daily dietary intake of Cd by adults in Poland is 11–30 µg, and in other countries it is 25–200 µg. The tolerable weekly intake of cadmium, taking into account the safety conditions and the degree of environmental contamination with the element, is set at 7 µg/kg body weight/week (Codex Alimentarius Commission, Rome, Italy, 1998). In adults, the safe threshold for cadmium intake is 51–71 µg/day [33]. According to the FAO/WHO recommendations, the tolerable consumption of Cd by an adult is about 0.4–0.5 mg/week, and the acceptable dose is 60–70 µg per day [33]. The estimated total mass of the element in an average human (with a body weight of 70 kg) is about 50 mg and increases with age [1,3,33].

The absorption of Cd from the gastrointestinal tract can be determined not only by the state of health but also by the diet, and thus the content of essential elements (mainly iron), vitamins, polyphenols, and antioxidants. A balanced diet that includes certain bioelements can prevent the absorption and toxic effects of Cd. At the same time, the deficiency of some biologically active substances may increase the absorption of Cd from the gastrointestinal tract and its accumulation in the human body [5,7,17]. Some studies indicate that Cd may affect bones and simultaneously cause a deficiency of calcium, protein, and vitamin D [5]. According to the EFSA, the intake of Cd in infants and young children is almost twice as much as in adults, as children’s dietary patterns (frequency and quantity) are often less diverse [65].

The presence of Cd in food can be related to the use of cadmium-plated dishes and galvanized equipment, cadmium-containing stabilizers in plastics, and cadmium-based pottery or glazes. A minimal amount of Cd can be found in drinking water due to the use of galvanized pipes and/or solders in tap fittings [5,8,9]. Jakubowski showed that the storage of food in enameled containers can lead to elevated Cd levels, especially in acidic liquids [75].

Cadmium is carried by dust or smoke over long distances and eventually falls to the ground, after which it easily accumulates in the soil and is introduced into the food chain following absorption by plants [5,73]. The accumulation of Cd in plants and edible parts of crops occurs through metabolic pathways of elements such as Zn and Fe [5,76]. Cd inhibits the growth of roots and shoots, decreases the content of chlorophyll and the rate of photosynthesis, causes leaf necrosis, and inhibits enzyme activity and many other processes, which ultimately limit the growth and development of plants [77]. It should be emphasized that the presence of Cd in food depends on the geographical location, bioavailability from the soil, genetics of crops, as well as applied agronomic practices, and consequently, postharvest reactions [8]. Microbial fermentation has also been shown to be one of the promising methods of Cd removal from food [9,78]. High concentrations of Cd have been found in cereals, especially whole grains, leafy vegetables (e.g., spinach), potatoes, and other root vegetables, as well as some seeds. Polluted aquatic environments also show increased Cd activity and accumulation of this element in select seafood, such as crustaceans and mollusks [4,5,9,79]. As much as 70–80% of the dietary Cd intake in humans is associated with food of plant origin [74]. Meanwhile, in red meat and fish products, the content of this element is relatively lower [80]. In the USA, about 300 food and drink products were analyzed, of which 10 products showed the highest mean lower-bound concentrations of cadmium: sunflower seeds (375 μg/kg), cooked spinach (117 μg/kg), potato chips (93 μg/kg), leaf lettuce (62 μg/kg), iceberg lettuce (54 μg/kg), peanut butter (53 μg/kg), shredded wheat cereal (51 μg/kg), dry roasted peanuts (45 μg/kg), French fries (44 μg/kg), and cooked liver (38 μg/kg) [81]. In their study, Clemens et al. found that vegetarians are characterized by a three-times-higher relative Cd intake than non-vegetarians [65]. Shao et al. demonstrated that Cd concentrations in rice samples were low (15.5 ± 16.0 μg/kg), which, according to the authors, is a safe level compared to the other tested samples [60]. In contrast, lower average Cd concentrations were detected in rice from Nepal (50 μg/kg) [82], Sri Lanka (80 μg/kg) [83], Malaysia (160 μg/kg) [84], India (19.1 μg/kg), and Thailand (13.0 μg/kg) [85]. Therefore, it is very important to monitor and constantly control the cadmium contamination of food products, mainly to ensure the safety of food supplies around the world [5,86].

## 7. Prevention and Monitoring of Cadmium Poisoning

Due to the toxic effects of Cd, people suffering from conditions such as kidney diseases, chronic bronchitis, emphysema, rhinitis, osteoporosis and osteomalacia, anemia, liver damage, and hypertension, or those who smoke regularly should not be employed in cadmium-exposed environments. Broadly defined preventive action should be undertaken to shape pro-health attitudes, focusing on promoting various positive health behaviors, including combating nicotinism and alcoholism, observing strict personal hygiene, and making employees aware of Cd exposure. Meanwhile, employers should limit Cd emission at workplaces to a level that does not exceed the daily concentration standards. In Poland, the current level of daily cadmium exposure is 0.02 mg/m^3^ from fumes and 0.04 mg/m^3^ from dust [3].

For acute oral poisoning, gastric lavage and disodium phosphate administration (4–8 g in a glass of water) are the recommended treatments, which work by binding to any Cd that has not been removed. However, chelating drugs are not recommended as they may cause kidney damage. On the other hand, chronic poisoning is treated symptomatically [3]. Cadmium pollution is mainly caused by massive vehicle traffic, the burning of fossil fuels, mining and metallurgical activities, and sediment removal. In order to mitigate the harmful health effects of cadmium, compounds such as polyphenols, melatonin, carotenoids, L-carnitine, and coenzyme Q10 can be used. Alternatively, the reduction of cadmium and other heavy metals can be achieved with the use of plants or nanoparticles that accumulate them by phytoremediation, which is relatively cheap, effective, and environmentally friendly [14,86]. Auxiliary tests, including complete blood count, the determination of prothrombin time, and index or liver function tests are useful for determining the concentration of Cd in the body of exposed persons [3].

Interestingly, despite guidelines for dietary Cd exposure limits in both Europe and the US, no such standards have been set in China [70].

## 8. Conclusions

Cadmium is one of the most toxic elements to which humans may be exposed at work or in the natural environment. Exposure to Cd is mainly through inhalation, food, and water, and after absorption, the element is retained in the body and accumulates throughout life (even up to 30 years). Cd can induce epigenetic changes that play a key role in the development of various cancers, chronic diseases, or other pathogenic disorders. Chronic exposure to Cd in humans may induce carcinogenesis. The other effects of Cd include oxidative stress and ROS production, which are normally counterbalanced by activating enzymatic (SOD, CAT, and GPx) and non-enzymatic (GSH, vitamin C, and vitamin E) barriers. It should be mentioned that providing adequate food with micronutrients (Zn, Fe, and Ca) can protect against the absorption and toxicity of cadmium. The continuous monitoring of individuals who are occupationally exposed to heavy metals such as cadmium is necessary to maintain a healthy lifestyle, as well as implement effective preventive measures and improve public health. Therefore, people exposed to Cd poisoning (e.g., metallurgists, mechanics, farmers, plumbers, and firemen) should regularly undergo a complete diagnostic assessment to determine the blood concentration and possible neurotoxicity of Cd.

## Figures and Tables

**Table 1:** Summary of Cd toxicity in individual parts of the human body [4,5,6,7,9,18,23,24,25,26,31,32,33,34,35,36,37,38,39,40,41,42,43,44].

Human Body	Effects of Exposure to Cadmium Poisoning
Respiratory system	– Irritation to the mucous membranes of the nose (disrupting the sense of smell) and the upper respiratory tract – Pulmonary edema – Chronic bronchitis – An impaired sense of smell (or even its complete loss) – Dry cough followed by expectoration – Dyspnea on exertion with reduced exercise tolerance and lung ventilation efficiency
Nephrological system	– Proteinuria – Glycosuria, aminoaciduria, increased excretion of calcium and phosphorus in the urine, and increased creatinine – Toxic nephropathy
Circulatory system	– Hypertension, atherosclerosis, and impaired heart function – Ischemic stroke
Skeletal system	– Bone demineralization, osteomalacia, and osteoporosis – Persistent pain in the spine, pelvis, and limbs – Itai-Itai disease (duck walking, pain in bones and joints); – Reduced efficiency of vitamin D metabolism and calcium absorption
Reproductive system	– Teratogenic effect-crosses the barrier to the fetus – Decreases the number of normal sperm forms – Impairs the function of the testes (damage to the vascular endothelium, Leydig and Sertoli cells, and intercellular connections, and necrosis of the seminiferous tubules, inhibition of testosterone synthesis, and impaired spermatogenesis) – Dysfunction of the prostate gland (changes in hormonal and secretory functions, impaired male fertility)
Nervous system	– Neurodegenerative diseases – Involvement in the etiopathogenesis of, e.g., AD, PD, HD, ALS, and MS – Cognitive and behavioral deterioration – In children, neurological, learning, and/or behavioral difficulties, neuropathy, and deterioration of memory, attention, and psychomotor functions

## Data Availability

Not applicable.

## Sample Availability

Samples of the compounds are not available from the authors.
